# Post-Acute Sequelae of COVID-19 and Cardiovascular Autonomic Dysfunction: What Do We Know?

**DOI:** 10.3390/jcdd8110156

**Published:** 2021-11-15

**Authors:** Giandomenico Bisaccia, Fabrizio Ricci, Vittoria Recce, Antonio Serio, Giovanni Iannetti, Anwar A. Chahal, Marcus Ståhlberg, Mohammed Yunus Khanji, Artur Fedorowski, Sabina Gallina

**Affiliations:** 1 Department of Neuroscience, Imaging and Clinical Sciences, Institute for Advanced Biomedical Technologies, “G. d’Annunzio” University of Chieti-Pescara, Via Luigi Polacchi, 66100 Chieti, Italy; giandomenico.bisaccia@studenti.unich.it (G.B.); vittoria.recce@studenti.unich.it (V.R.); antonio.serio@studenti.unich.it (A.S.); sgallina@unich.it (S.G.); 2 Department of Clinical Sciences, Lund University, 20502 Malmö, Sweden; artur.fedorowski@med.lu.se; 3 Casa di Cura Villa Serena, Città Sant’Angelo, 66013 Pescara, Italy; 4 Department of Translational and Precision Medicine, Sapienza University of Rome, 00185 Rome, Italy; iannetti.1752004@studenti.uniroma1.it; 5 Center for Inherited Cardiovascular Diseases, WellSpan Health, 140 North Pointe Boulevard, Lancaster, PA 17601, USA; Anwar.Chahal@pennmedicine.upenn.edu; 6 Division of Cardiology, Department of Medicine, University of Pennsylvania, 3400 Spruce Street, Philadelphia, PA 19104, USA; 7 Department of Cardiovascular Diseases, Mayo Clinic, 200 First St. SW, Rochester, MN 55905, USA; 8 Barts Heart Centre, Barts Health NHS Trust, London EC1A 7BE, UK; m.khanji@qmul.ac.uk; 9 Cardiology, Heart, Vascular and Neuro Theme, Karolinska University Hospital, Department of Medicine, Solna, Karolinska Institutet, 17177 Stockholm, Sweden; marcus.stahlberg@ki.se; 10 Department of Cardiology, Newham University Hospital, Barts Health NHS Trust, London E13 8SL, UK; 11 NIHR Barts Biomedical Research Centre, William Harvey Research Institute, Queen Mary University of London, London EC1A 7BE, UK

**Keywords:** COVID-19, cardiovascular autonomic dysfunction, long COVID syndrome, PASC, postural orthostatic tachycardia, post-acute sequelae, autoimmunity

## Abstract

Post-acute sequelae of SARS-CoV-2 (PASC), or long COVID syndrome, is emerging as a major health issue in patients with previous SARS-CoV-2 infection. Symptoms commonly experienced by patients include fatigue, palpitations, chest pain, dyspnea, reduced exercise tolerance, and “brain fog”. Additionally, symptoms of orthostatic intolerance and syncope suggest the involvement of the autonomic nervous system. Signs of cardiovascular autonomic dysfunction appear to be common in PASC and are similar to those observed in postural orthostatic tachycardia syndrome and inappropriate sinus tachycardia. In this review, we report on the epidemiology of PASC, discuss current evidence and possible mechanisms underpinning the dysregulation of the autonomic nervous system, and suggest nonpharmacological and pharmacological interventions to treat and relieve symptoms of PASC-associated dysautonomia.

## 1. Introduction

The coronavirus disease 2019 (COVID-19) pandemic was declared a global health emergency in early 2020 and has since affected over 217 million people worldwide, causing the death of more than 4.5 million by 1 September 2021 [1]. The long-term effects of severe acute respiratory syndrome coronavirus 2 (SARS-CoV-2) infection are largely unknown, yet beyond the first 30 days of illness, COVID-19 survivors are reportedly at higher risk of death, health care resource utilization, and exhibit a broad array of pulmonary and extrapulmonary clinical manifestations, including neurologic and cardiologic disorders, and a spectrum of symptoms related to poor general wellbeing, even after the resolution of the acute illness [2]. Since the publication of preliminary reports concerning post-COVID syndrome, a plethora of definitions have been proposed. The term “post-acute sequelae of SARS-CoV-2 infection” (PASC), otherwise referred to as “long COVID” or “long-haul COVID”, refers to persistent and prolonged effects after acute COVID-19 [3,4] and has been officially accepted by the National Institutes of Health [5] to describe the persistence of symptoms or development of sequelae beyond 3 weeks from the onset of acute symptoms of SARS-CoV-2 infection. The term “chronic COVID”, on the other hand, has been proposed [6] for patients presenting with ongoing symptoms beyond 12 weeks. Symptoms characterizing PASC include fatigue, dyspnea, post-exertional malaise, chest pain, and palpitations [7,8]. Concerns have been raised that PASC might be the clinical expression of COVID-related cardiovascular autonomic dysfunction [9], with some overlapping features suggestive of postural orthostatic tachycardia syndrome (POTS) [10]. In this review, we (1) discuss the most recent evidence on PASC-related dysautonomia and (2) provide possible pathways leading to cardiovascular autonomic dysfunction (CVAD) after SARS-CoV-2 infection.

## 2. Post-Acute Sequelae of COVID-19

The idea that SARS-CoV-2 infection could be associated with long-term health outcomes first appeared in the literature in mid-2020 [11], along with previous evidence of long-haul symptoms following the Ebola [12] virus outbreak and infections from previous coronaviruses [13,14]. COVID-19 survivors have been shown to experience a constellation of symptoms such as fatigue, dyspnea or breathlessness [8,15], palpitations [16,17], brain fog, lack of concentration [18,19], sleep disturbances (i.e., insomnia) [20], headache [17,21,22,23,24,25], orthostatic intolerance [25], anxiety and post-traumatic stress disorder [20,23,26,27,28], chest pain, joint pain [8], sore throat [17], and hair loss [15] persisting >4 weeks after recovery. These clinical sequelae share similarities with post-acute symptoms reported after severe acute respiratory syndrome (SARS) and Middle East Respiratory Syndrome (MERS) epidemics, caused by previous coronaviruses [14]. Such heterogeneous postmorbid clinical presentation, with a plethora of specific signs and symptoms often being interpreted as non-physiologic or related to mental-health-related conditions, lends itself to the potential marginalization of patients [28].

Reports on the persistence of symptoms after COVID-19 appeared in the literature as early as April 2020. Notably, patients in good general health before infection, with minimal or no comorbidities, started experiencing prolonged symptoms and cyclic recurrences. In a cohort of 143 Italian COVID-19 survivors, 55% still experienced ≥3 symptoms two months after disease onset, and 44% reported their quality of life was significantly worsened [8]. The most frequent symptoms described in this cohort included fatigue, dyspnea, joint pain, and chest pain. Similar findings were observed in another cohort of COVID-19 survivors demonstrating that the presence of severe fatigue was not related to disease severity in the acute phase of the illness [27]. In a longitudinal study conducted in the Faroe Islands, of 180 COVID-19 outpatients, 53% reported the persistence of ≥1 symptom four months after the disease onset [29]. In another cohort of 227 Spanish COVID-19 survivors, the persistence of symptoms was still recorded in >50% of patients three months after their illness [30]. Similarly, about 70% of patients enrolled in the post-hospitalization COVID-19 study (PHOSP-COVID) reported ongoing symptoms five months after discharge [31].

Fatigue is by far the most commonly reported symptom among subjects with previous COVID-19 infection. Severe fatigue is more frequently reported by female and younger patients [32], and was associated with a high prevalence of anxiety [33] and post-traumatic stress disorder. Notably, fatigue described by PASC patients shows some distinct features from chronic fatigue syndrome (CFS). Studies on CFS patients showed impaired cerebral blood flow [34] and reduced heart rate variability [35,36], although these findings could not be confirmed in PASC patients [33]. Furthermore, less than 50% of patients with PASC-related fatigue met the diagnostic criteria for CFS [37]. This suggests that PASC is not just a COVID-19-related manifestation of CFS but a specific pathophysiological entity with a specific CVAD phenotype.

### How Long Will Symptoms Last?

This is currently unknown, although results of ongoing prospective studies should provide some answers. Contemporary reports show the high prevalence of symptoms several months after disease onset [38]. When reviewing the literature that explores the long-term effects of previous coronavirus epidemics, such as SARS, an overall reduction in quality of life was noted up to 1 year after infection, with the most commonly persisting symptoms being fatigue and exercise limitation [14]. 

There is growing concern that children and young people may also develop PASC [17,39]. Current evidence indicates that symptom patterns are similar to those seen in adults [40], although they occur less frequently in pediatric and younger adult populations, with the exception of fatigue [41] and insomnia [39]. Data from the Children and young people with Long COVID (CLoCk) UK study, collected at a time when the Alpha variant was predominant, indicate that young COVID-19 survivors reported higher rates of persistent symptoms, such as tiredness and headache, compared with matched individuals, regardless of symptoms experienced in the acute phase of infection [42].

## 3. COVID-19 Infection and Dysautonomia

A growing body of evidence links PASC to CVAD, with a number of reports suggesting the possibility of previously infected COVID-19 patients experiencing symptoms suggestive of POTS [10,13,16,43,44,45,46]. This is also consistent with previous evidence hinting at the presence of CVAD in SARS survivors during the 2003 epidemic [47].

It should be emphasized that POTS is a rare medical condition, and the onset of tachycardia following SARS-CoV-2 or any other viral infection can be more frequently explained as a physiological response of a perfectly intact autonomic nervous system. It has been recently suggested that post-COVID-19 tachycardia syndrome would represent a distinct disease entity, different from classical POTS [48].

Possible causes of tachycardia in COVID-19 survivors may include systemic conditions, deconditioning, anemia, hypoxia, anxiety, persisting fever, lung or cardiac disease, including sinus node dysfunction, myocarditis, and heart failure. Accordingly, comprehensive clinical workup and the appropriate use of cardiovascular and autonomic tests are mandatory to ensure accurate diagnosis (for more information, see Section 7, “Diagnosis of post-COVID dysautonomia”).

Estimation of the true prevalence and assessment of clinical features of CVAD in PASC patients can be challenging for many reasons: firstly, prognostic studies designed to measure clinical outcomes in COVID-19 survivors do not generally allow for the assessment of prevalent or incident dysautonomia, as this would require the rapid implementation of specific autonomic tests that are not part of clinical routine and/or not generally available [49], particularly during the pandemic [11,46]; secondly, heterogeneity in the timing of clinical evaluation may hide time-specific disease features (Table 1 and Table 2); third, current reports focused on post-COVID CVAD cannot be compared to previous literature because data on autonomic dysfunction following SARS and MERS-CoV infections are scarce.

There is some convincing evidence about the presence of CVAD-related symptoms in PASC patients. In a cohort of 179 COVID-19 survivors, palpitations and dizziness were reported in 20% of cases after eight-month follow-up, while headache and concentration problems were reported by 40% of participants [23]. Palpitations were reported in 7% of cases two weeks after acute COVID-19 in Nigerian survivors [22], in 9% of cases from a Chinese cohort of 1733 patients followed-up at 6 months from disease onset [53], and in 23% of cases from a multi-center UK cohort [38]. In the latter study, fatigue was reported by 82% of respondents, shortness of breath by 54%, sleep disturbances by 46%, headache by 39%, and dizziness or lightheadedness by 36%. 

Fatigue is a relatively common feature in POTS [49] and is usually associated with other signs of dysautonomia, as seen in PASC patients [13]. Recent evidence showed the significant burden of fatigue, symptoms of autonomic dysfunction, and mood disorders in the aftermath of COVID-19 infection, but reassuringly did not demonstrate pathological findings during investigations for autonomic function [33]. Fatigue is linked to the higher prevalence of anxiety and post-traumatic stress disorder in COVID-19 survivors [23,27,33,54], while somatic anxiety in patients with chronic fatigue has been directly associated with autonomic nervous system dysfunction [55].

**Table 2:** Case series of patients diagnosed with CVAD following COVID-19.

Authors, Year	Dani et al. [16], 2021	Johansson et al. [10], 2021	Blitshteyn et al. [56], 2021	Shouman et al. [25], 2021	Wallukat et al. [57], 2021	Goodman et al. [58], 2021	Overall
Sample size	5	3	20	20	7	6	61
Age	43	36	42	44	45	45	42
Female sex	100%	33%	70%	70%	57%	67%	69%
CVAD (*n*)	OI (4) rTC (1)	POTS (3)	POTS (15), NCS (3), OH (3)	OH (14) POTS (6)	POTS (7)	POTS (4), OH (1), PHTN (3)	POTS 69% of cases
Palpitations, tachycardia	60%	67%	80%	15%	57%	67%	58%
Fatigue	60%	67%	60%	55%	43%	100%	64%
Vertigo ^a^	40%	67%	25%	100%	0%	100%	55%
Dyspnea	20%	67%	45%	45%	0%	83%	43%
Presyncope	20%	67%	15%	5%	0%	83%	32%
Chest pain	20%	67%	15%	25%	0%	50%	30%
Headache	0%	67%	15%	40%	0%	67%	31%
Brain fog ^b^	0%	67%	5%	25%	14%	0%	19%
Sleep disturbances	0%	67%	0%	20%	0%	0%	15%

CVAD, cardiovascular autonomic dysfunction; NCS, non-cardiogenic syncope; OH, orthostatic hypotension; OI, Orthostatic intolerance; PHTN, postural hypertension; POTS, postural orthostatic tachycardia syndrome; rTC, reactive tachycardia. All patients were assessed by clinical evaluation, except for one (Dani et al.) who was interviewed by phone call. Exact assessment timing was not reported in Dani et al., Wallukat et al. Among all studies that reported it, mean time at assessment was 4–5 months after acute COVID-19. Among all studies, POTS was diagnosed in 69% of patients. The study by Townsend et al. [33] was not included in this table for lack of information concerning the patients’ symptoms and diagnoses. ^a^ includes all patients reporting vertigo, lightheadedness, and/or dizziness. ^b^ includes all patients reporting brain fog and/or difficulty concentrating.

### When Does CVAD Occur in COVID-19 Patients?

Current evidence seems to indicate that signs of CVAD, including relative bradycardia, first appear during the acute phase of illness. A study of 63 COVID-19 patients and 43 matched healthy controls showed increased parasympathetic tone during acute infection, with no significant difference between symptomatic and asymptomatic patients [59]. Moreover, dysautonomia has been reported as the main neurological manifestation in approximately 2% of acute COVID-19 cases [60]. 

The first two case reports on CVAD onset during SARS-CoV-2 infection concerned elderly patients. One case was of a 70-year-old lady hospitalized due to recurrent syncope and non-epileptic seizures, who tested positive for SARS-CoV-2 on the same day [61]. The second case involved a 72-year-old male with COVID-19 who presented with a syndrome characterized by highly labile blood pressure readings, with hypertensive crises alternating with hypotensive episodes explained by acute dysautonomia due to afferent baroreflex failure [62]. Other reports concerned COVID-19 patients presenting with orthostatic hypotension, syncope, and other CVAD clinical manifestations [43,45].

There is current uncertainty regarding PASC-related dysautonomia in the pediatric population. In a case series, five children (80% females) aged between 9 and 15 years old had been complaining of severe fatigue and palpitations for eight months after acute infection [17] but did not undergo any autonomic investigation. In another case series of three children with confirmed or probable COVID-19, the early development of symptoms of orthostatic intolerance led to a subsequent diagnosis of POTS during a passive standing test performed 5–7 months after the onset of the infection [41].

## 4. Postural Orthostatic Tachycardia Syndrome after COVID-19

Beyond appropriate sinus tachycardia, which comprises both physiological and pathologic causes of increased heart rate, a possible explanation for palpitations and fatigue in COVID-19 survivors is POTS, which is a dysautonomia characterized by an excess in heart rate increase on standing and orthostatic intolerance [49]; it is more frequent in females (80% of cases), and the young (especially 15–45 years old). POTS is misdiagnosed in up to 75% of patients and viral infection is recognized as a trigger in up to 41% of cases [63].

In March 2021, the American Autonomic Society (AAS) issued a statement concerning the clinical overlap of PASC with POTS [4]. The AAS stated that most PASC symptoms can be suggestive of POTS when the patient also suffers from excessive orthostatic tachycardia, and that further, in-depth studies are needed to elucidate the duration of post-COVID CVAD, its underlying pathophysiology, and optimum treatment.

There is increasing evidence of incident cases of postural tachycardia following COVID-19. In an international online survey on 802 COVID-19 survivors who received a post-acute diagnosis, 19% reported receiving a POTS diagnosis [54]. In this cohort, POTS was the second most common reported diagnosis in the aftermath of COVID-19, following migraine (27%).

The first case of POTS following acute SARS-CoV-2 infection concerned a 26-year-old lady with no history of CVAD [50]. The patient showed excessive postural tachycardia and was diagnosed with POTS during hospitalization; while respiratory symptoms resolved after about 3 weeks, hyperadrenergic symptoms linked to sympathetic over-compensation lasted for several months after the initial infection. 

Following this, a number of cases were published describing CVAD occurring weeks or months after COVID-19. A 36-year-old woman who recovered from symptomatic SARS-CoV-2 infection in self-isolation started experiencing fatigue, headache, dizziness, chest pain, and orthostatic palpitations one month after the onset of acute infection [44] and was later diagnosed with POTS upon head-up tilt testing. Table 1 summarizes a list of cases reporting on persisting signs and symptoms of CVAD following COVID-19.

## 5. How Does SARS-CoV-2 Infection Cause Long-Term Dysautonomia?

Infections are known triggers of dysautonomia, particularly in POTS. Among various mechanisms by which SARS-CoV-2 may induce dysautonomia, three possible conditions have been proposed and gained support from preliminary evidence: hypovolemia, brainstem involvement, and autoimmunity [64]. 

**Figure 1:**
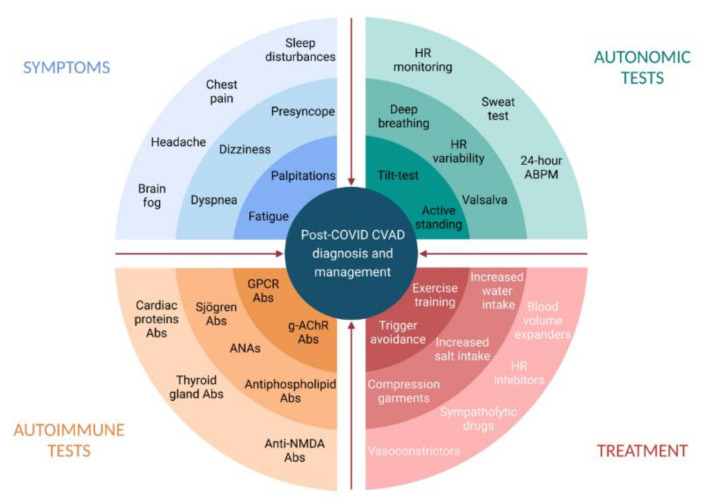
Post-COVID cardiovascular autonomic dysfunction: diagnosis and management. Abs, antibodies; ANAs, antinuclear antibodies; CVAD, cardiovascular autonomic dysfunction; g-AChR, ganglionic neuronal nicotinic acetylcholine receptor; GPCR, G-Protein coupled receptor; HR, heart rate. Created with BioRender.com.

Hypovolemia has been widely recognized in PASC patients with dysautonomic features [25,52]. Hypovolemia may trigger hyperadrenergic POTS and lead to cerebral hypoperfusion and the impairment of central autonomic networks [56]. Preliminary evidence on post-COVID POTS supports the correction of hypovolemia by the liberal intake of water and salt [16] and other nonpharmacological interventions aimed at intravascular volume expansion and increasing venous return [65] (Figure 1).

Brainstem dysfunction can be associated with any clinical manifestation of PASC [66], including autonomic failure [67]. Proposed mechanisms for brainstem dysfunction following SARS-CoV-2 infection include direct viral invasion, neuroinflammation, vascular activation [66], and brainstem compression [56]. The nucleus tractus solitarii and reticular activation system have been involved in pathogenic hypotheses. Notably, a mild grade of brainstem dysfunction has been consistently demonstrated via magnetic resonance imaging in some conditions overlapping and comorbid to POTS, such as chronic fatigue syndrome [68]. Brainstem dysfunction following COVID-19 has been found to be similar to that observed in takotsubo syndrome (TTS) [64], and interestingly, a case for COVID-19-related TTS has recently been described [69]. Although excessive endogenous stress hormone responses in the general population could likely explain the increased incidence of TTS during the pandemic [70,71], lesions of the brainstem, which has autonomic centers, have also been hypothesized as neural substrates [72].

There is evidence suggesting a major role for autoimmunity in the pathophysiology of post-viral POTS [73]. Patients with POTS have been shown to have a higher prevalence of specific autoantibodies (AAbs), including G-Protein coupled receptor (GPCR) antibodies, which could explain an increase in sympathetic tone by activating adrenergic receptors and eliciting a negative allosteric effect on muscarinic GPCRs [74,75]. These serum patterns have been confirmed in COVID-19 survivors with dysautonomia [57] and are comparable to those seen in patients with CFS and small-fiber neuropathy [56]. A severe orthostatic intolerance syndrome was reported in a young lady with previous COVID-19 infection who was later diagnosed with small-fiber neuropathy and who responded well to the administration of intravenous immunoglobulins, thus suggesting an autoimmune trait likely caused her symptoms [51]. AAbs may trigger autonomic symptoms by activating adrenergic and cholinergic receptors and/or inducing peripheral vasodilation [49]. In a case series of 31 patients suffering from different long COVID-19 symptoms, an unusually high number of functionally active AAbs targeting GPCR with chronotropic and vasoactive functions was detected in the sera of these patients [57]; the specific AAbs pattern identified has previously been observed in other neurological and cardiac conditions and might explain the development of PACS. Other recognized AAbs in POTS include circulating anti-nuclear, anti-thyroid, anti-cardiac protein, anti-phospholipid, and Sjögren’s antibodies. Notably, antiphospholipid antibodies were detected in a patient with PASC-related POTS [60] who developed clinical signs and symptoms of antiphospholipid syndrome and mast cell activation syndrome following SARS-CoV-2 infection. 

The autoimmune hypothesis can also explain the higher prevalence of symptoms in women, as prolonged estrogen exposure and infections are known to synergistically trigger autoimmunity [76,77].

Another possible explanation of coronavirus-induced CVAD is the direct damage of extracardiac postganglionic sympathetic neurons by the virus [64]. However, there is currently no convincing evidence to support this hypothesis.

Finally, the role of excessive mast cell activation in the pathophysiology of POTS has been proposed. In mast cell activation disorder, the inappropriate release of histamine and other mast cell mediators in response to physical activity or orthostatic stress may lead to orthostatic tachycardia [49]. Flushing, headaches, and gastrointestinal symptoms are more prevalent in these individuals, and a recent study showed markedly elevated prostaglandins and histamine markers in patients with gastrointestinal complaints, reinforcing the case for a mast cell activation disorder in a subset of POTS patients [78]. A case of mast cell activation syndrome was described in a 50-year-old female with previous COVID-19 with signs and symptoms of POTS [52]. Pharmacological treatment and non-pharmacological measures against POTS and mast cell activation syndrome gradually improved her symptoms, despite the patient continuing to test positive for POTS by active testing more than one year after onset of COVID-19 infection.

## 6. Is PASC Gender-Specific?

Data from a large retrospective cohort study in the UK showed that patients admitted to the hospital for COVID-19 were more frequently male [79]. Males also showed higher mortality [15]. Conversely, COVID-19-related multi-organ dysfunction occurred in a gender-neutral fashion, with both male and female COVID-19 survivors showing a similar risk of respiratory and CV complications [79]. In a cross-sectional study, survivors experiencing symptoms such as fatigue six weeks after the onset of infection were typically females [27]. At five months from disease onset, among 1077 survivors in the PHOSP-COVID study, middle-aged females were more likely to experience long-haul symptoms [31,80]. In another study from the UK, it was shown that women aged below 50 years were 5 times less likely to be hospitalized for acute COVID-19, but had twice the risk of experiencing long term symptoms after seven months of follow-up [38]. According to a report from Nigeria, male subjects were at a lower risk of persistent symptoms following SARS-CoV-2 infection [22], whereas a study from Saudi Arabia did not confirm these findings [81]. In the CLoCK study that enrolled a cohort of children and young people aged 11–17, females more frequently reported multiple symptoms compared with males both at PCR-testing and 3-month follow-up [42].

### Why Would Women Be at Higher Risk of Developing Post-COVID-19 Symptoms?

A single explanation is not currently available. The role of hormonal dysregulation has been proposed, but PASC can also be the result of autoimmunity [39], and, although less susceptible to infectious disease compared with men, women are more often prone to autoimmune diseases [39,76]. Male patients showed higher COVID-19-related mortality, while the female sex is overrepresented among symptomatic COVID-19 survivors [38]. Nevertheless, disease severity has not been shown to predict subsequent long COVID syndrome [27].

In a case series of 20 long COVID patients (70% women), 75% presented with POTS, followed by orthostatic hypotension and neurocardiogenic syncope [82]. From the analysis of case series currently available in the literature, the female sex accounts for roughly 70% of PASC cases with various dysautonomic features (Table 2). In particular, middle-aged women experience the most severe, long-lasting symptoms after being treated in hospital for COVID-19. Consistently, all published case reports, with the exception of one, concerned women (Table 1). This trend can be also observed in the pediatric population [17].

## 7. Diagnosis of Post-COVID Dysautonomia

The diagnosis of post-COVID dysautonomia requires the comprehensive assessment of previous medical history, the careful evaluation and characterization of symptoms, and expert interpretation of neurological and cardiovascular autonomic investigations [4,16,83].

Patients’ medical histories and their recollection of symptoms are essential in detecting the presence of dysautonomia [84]. The histories of SARS-CoV-2 survivors with persistent autonomic dysfunction may reveal frequent fainting episodes, dizziness, lightheadedness, and/or palpitations [63] prior to SARS-CoV-2 infection, revealing underlying hypotensive susceptibility or previous orthostatic intolerance syndromes. Frequent comorbidities include migraine, irritable bowel syndrome, chronic fatigue syndrome, and autoimmune diseases [73,77]. Lightheadedness, tachycardia, dyspnea, headache, and poor concentration are the most frequent symptoms in PASC patients. Fatigue is the single most frequent symptom, although it is not specific for dysautonomia [33]. Severe symptoms may last for weeks or even months after infection and negatively affect overall quality of life [23].

In post-COVID patients with suspected dysautonomia, the Composite Autonomic Symptom Scale 31 (COMPASS-31) questionnaire can be a sensitive tool to test the likelihood of autonomic dysfunction [85]. This questionnaire has been previously implemented in COVID-19 survivors, showing significantly higher scores than controls [86] with an optimal cut-point for rule-out of CVAD of 13.25 [86,87].

The differential diagnosis of fatigue, dyspnea, palpitations and sinus tachycardia should be carefully scrutinized in order to identify and treat common medical conditions overlapping with long COVID illness. Blood tests (including complete blood count, renal function, B-type natriuretic peptide, electrolytes, thyroid stimulating hormone, and morning cortisol), resting 12-lead ECG, supine and upright blood pressure, and the 6-min walking test should be routinely evaluated [88]. Cardiothoracic imaging (chest X-ray, chest CT, echocardiography, and cardiac magnetic resonance) and exercise testing are invaluable diagnostic tools in investigating PACS and COVID-19 complications.

**Figure 2:**
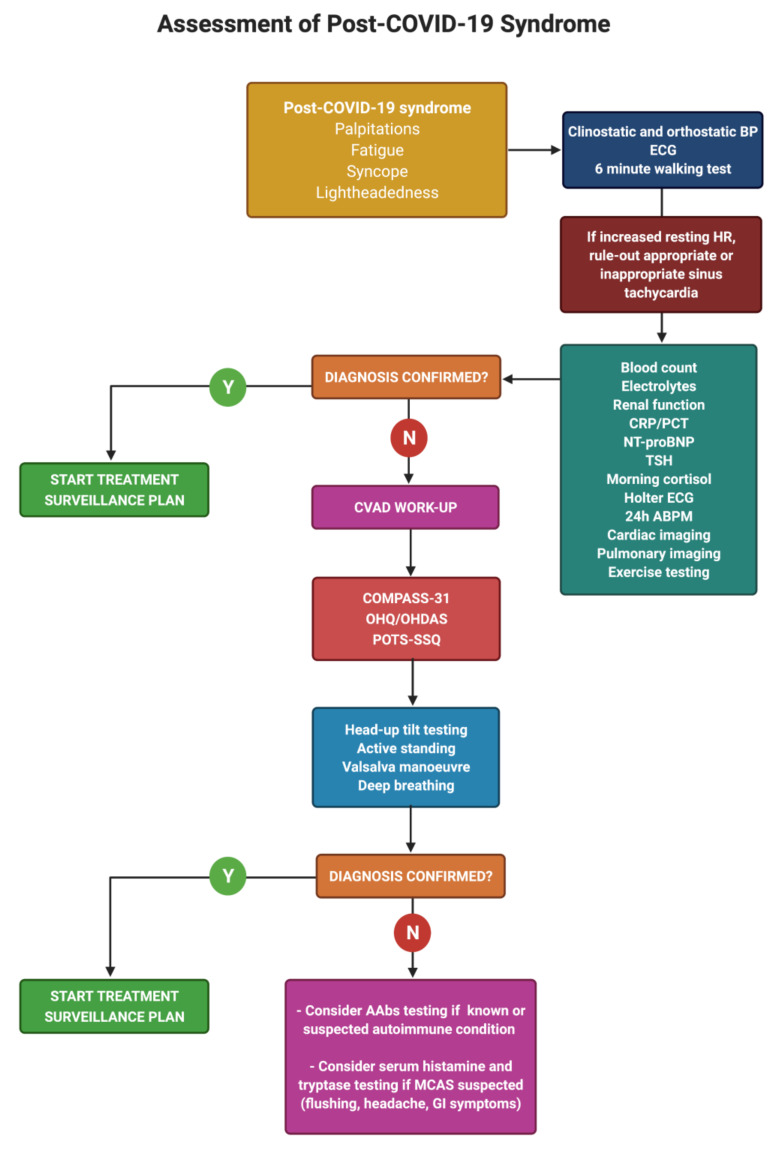
Assessment of post-COVID-19 Syndrome. AAbs, autoantibodies; ABPM, ambulatory blood pressure monitoring; BP, blood pressure; COMPASS-31, Composite Autonomic Symptom Scale 31; CRP, C-reactive protein; CVAD, cardiovascular autonomic dysfunction; ECG, electrocardiogram; GI, gastrointestinal; MCAS, mast cell activation syndrome; NT-proBNP, N-terminal prohormone of brain natriuretic peptide; OHDAS, orthostatic hypotension daily activity scale; OHQ, orthostatic hypotension questionnaire; PCT, procalcitonin; POTS-SSQ, postural orthostatic tachycardia syn-drome symptom scoring questionnaire, as in Johannsson and colleagues [10]; TSH, thyroid stimu-lating hormone. Created with BioRender.com.

When cardiovascular autonomic dysfunction is suspected, autonomic tests can be indicated to confirm the diagnosis (Figure 2).

Active standing and/or head-up tilt tests can be very useful in evaluating PASC patients (Table 3), especially among individuals with inappropriate/orthostatic tachycardia, unexplained syncope, or syndromes of orthostatic intolerance [88,89]. Other autonomic investigations include 24 h ambulatory blood pressure monitoring and ECG monitoring [16,33], heart rate variability, Valsalva maneuver, deep breathing [58], and sweat function testing [25,90].

PASC patients with POTS-like symptoms feature a high prevalence of circulating AAbs, at times with peculiar patterns [57] (Figure 1). Although neither sensitive nor specific, AAbs testing can be helpful in selected cases. Specific tests for mast cell activation syndrome can be also considered in PASC patients with flushing episodes, frequent headaches, and persistent gastrointestinal symptoms.

## 8. Management of Post-COVID Dysautonomia

Once a diagnosis of CVAD is obtained, several treatment options are available, although specific disease-modifying therapies are still limited and represent an unmet need [49].

Non-pharmacological measures should be considered as first-line treatment options and include physical reconditioning with aerobic progressive exercise training programs, the use of compression garments, the liberal intake of water and salt, drinking water before getting up in the morning, sleeping with the head of the bed elevated, the careful avoidance of situations that can exacerbate symptoms (sleep deprivation, heat exposure, alcohol intake, or large or heavy meals) [65,91]. Physical maneuvers such as leg crossing, muscle tensing, and squatting have been shown to be effective in delaying/preventing vasovagal syncope if used at the onset of prodromal symptoms.

Pharmacological therapies have been frequently used in PASC patients (Table 3). These should be reserved for patients which do not respond to nonpharmacological therapies and are complementary to nonpharmacological measures in patients with severe, refractory symptoms. Recommended drugs have also been extensively used where symptoms persist [10,16]. These include volume expanders (fludrocortisone, desmopressin, and intravenous saline), heart rate inhibitors (propranolol, ivabradine, and pyridostigmine), vasoconstrictors (midodrine, octreotide, methylphenidate, and droxidopa) and sympatholytic drugs (clonidine and methyldopa) [10,16].

Decisions regarding which treatment to initiate should be guided by specific symptoms and hemodynamic patterns, i.e., tachycardic vs. hypotensive phenotypes. 

The tachycardic phenotype can be treated with beta-blockers, i.e., metoprolol, or ivabradine [88]. Recently, intravenous metoprolol has been tested for use in the treatment of acute respiratory distress in acute COVID-19 and was found to improve oxygenation and reduce alveolar inflammation, shortening the duration of invasive mechanical ventilation overall [92]. Large-scale randomized evidence is eagerly awaited to confirm alleged pleiotropic anti-inflammatory effects of metoprolol [93] beyond its established safety, efficacy, and indications [94].

In patients with the hypotensive phenotype, droxidopa, midodrine, or pyridostigmine may be considered. In hypovolemic patients, intravenous saline infusion and intravascular volume expansion would be highly desirable, while the use of fludrocortisone and desmopressin should be reserved to patients with severe refractory symptoms. Sympatholytic drugs, such as clonidine and methyldopa, can be proposed to patients with hyperadrenergic features, including hyperhidrosis and tachycardia [46].

Beyond these drugs, immunological therapy with intravenous immunoglobulins has been proposed for compassionate use in a patient with autoimmune features [51], leading to the regression of brain fog and the improvement of fatigue and headache. However, further studies are needed to test the rationale, safety, and efficacy of specific immunomodulators/immunosuppressant agents in PASC patients with refractory CVAD-related symptoms.

## Figures and Tables

**Table 1:** Case reports of patients diagnosed with CVAD following COVID-19.

Authors, Year	Miglis et al. [50], 2020	Kanjwal et al. [44], 2020	Novak et al. [51], 2020	Umapathi et al. [46], 2020	Schofield et al. [52], 2021
Follow-up (months)	5.5	1	3	2.5	12
Mean age (years)	26	36	64	39	50
Sex	F	F	F	M	F
Diagnosis	POTS, OH	POTS	OCHOS	POTS	POTS
Palpitations, tachycardia	Yes	Yes	-	Yes	-
Fatigue	Yes	Yes	Yes	-	-
Vertigo ^a^	Yes	Yes	-	-	-
Dyspnea	Yes	-	-	-	Yes
Presyncope	Yes	-	-	-	Yes
Chest pain	Yes	Yes	-	-	Yes
Headache	-	Yes	Yes	-	-
Brain fog ^b^	Yes	-	Yes	-	-
Sleep disturbances	Yes	-	-	-	-

OCHOS, orthostatic cerebral hypoperfusion syndrome; OH, orthostatic hypotension; POTS, postural orthostatic tachycardia syndrome. All patients were subject to clinical observation. Patients’ previous medical history included exercise-induced asthma, eczema, diffuse urticaria, gastroesophageal reflux disease, hypertension, thyroid disease, diabetes mellitus, headache and/or episodic migraine, Lyme disease, obsessive compulsive disorder, small fiber neuropathy and orthostatic cerebral hypoperfusion syndrome. One patient not reporting significant medical history was described by Kanjwal et al. [44]. ^a^ includes all patients reporting vertigo, lightheadedness, and/or dizziness. ^b^ includes all patients reporting brain fog and/or difficulty concentrating.

**Table 3:** COVID-19 related CVAD: overview of diagnostic modalities and strategies of treatment.

Study Features		Autonomic Testing Modality						Treatment Strategy				
Authors, year	Study type	HUT	Active standing	Deep breathing	Valsalva	Sudomotor test	Holter monitoring	NPT ^a^	Volume expanders ^b^	HR Inhibitors ^c^	Sympatholytic Drugs ^d^	IT ^e^
Miglis et al. [61], 2020	Report	Yes	-	-	Yes	-	-	-	-	Yes	Yes	-
Kanjwal et al. [42], 2020	Report	Yes	-	-	-	-	-	Yes	-	Yes	-	-
Novak et al. [73], 2020	Report	Yes	-	Yes	Yes	Yes	-	-	-	-	-	Yes
Umapathi et al. [44], 2020	Report	Yes	Yes	-	-	-	-	Yes	Yes	Yes	-	-
Schofield et al. [63], 2021	Report	-	Yes	-	-	-	-	N.A.				
Dani et al. [16], 2021	Series	Yes	Yes	-	-	-	Yes	N.A.				
Townsend et al. [32], 2021	Series	-	Yes	Yes	Yes	Yes	Yes	N.A.				
Johansson et al. [10], 2021	Series	Yes	Yes	-	Yes	-	-	Yes	-	Yes	-	-
Blitshteyn et al. [77], 2021	Series	Yes	Yes	-	-	-	-	Yes	Yes	-	-	-
Shouman et al. [25], 2021	Series	Yes	-	-	Yes	Yes	-	Yes	-	Yes	-	-
Goodman et al. [80], 2021	Series	Yes	-	Yes	Yes	Yes	-	N.A.				

HR, heart rate; HUT, head-up tilt test; IT, immunotherapy; NFT, non-pharmacological therapy. The case series by Wallukat et al. (#69) is not included in this table, since neither specific testing nor therapy for patients with a diagnosis of dysautonomia were reported. Schofield et al., Dani et al., Townsend et al., and Goodman et al. did not report specific treatment. ^a^ Non-pharmacological therapy includes exercise training, withdrawal of exacerbating drugs, increased water intake, increased salt intake, and use of compressive garments; ^b^ volume expanders include fludrocortisone, desmopressin, and acute or chronic use of intravenous saline solution; ^c^ HR inhibitors include propranolol, ivabradine, and pyridostigmine; ^d^ sympatholytic drugs include clonidine and methyldopa; ^e^ immunotherapy consists of intravenous administration of immunoglobulins.

## Data Availability

Not applicable.
